# Validation of human microRNA target pathways enables evaluation of target prediction tools

**DOI:** 10.1093/nar/gkaa1161

**Published:** 2020-12-10

**Authors:** Fabian Kern, Lena Krammes, Karin Danz, Caroline Diener, Tim Kehl, Oliver Küchler, Tobias Fehlmann, Mustafa Kahraman, Stefanie Rheinheimer, Ernesto Aparicio-Puerta, Sylvia Wagner, Nicole Ludwig, Christina Backes, Hans-Peter Lenhof, Hagen von Briesen, Martin Hart, Andreas Keller, Eckart Meese

**Affiliations:** Chair for Clinical Bioinformatics, Saarland University, 66123 Saarbrücken, Germany; Institute of Human Genetics, Saarland University, 66421 Homburg, Germany; Department of Bioprocessing & Bioanalytics, Fraunhofer Institute for Biomedical Engineering, 66280 Sulzbach, Germany; Institute of Human Genetics, Saarland University, 66421 Homburg, Germany; Center for Bioinformatics, Saarland Informatics Campus, Saarland University, 66123 Saarbrücken, Germany; Chair for Clinical Bioinformatics, Saarland University, 66123 Saarbrücken, Germany; Chair for Clinical Bioinformatics, Saarland University, 66123 Saarbrücken, Germany; Chair for Clinical Bioinformatics, Saarland University, 66123 Saarbrücken, Germany; Institute of Human Genetics, Saarland University, 66421 Homburg, Germany; Chair for Clinical Bioinformatics, Saarland University, 66123 Saarbrücken, Germany; Department of Genetics, Faculty of Science, University of Granada, 18071 Granada, Spain; Instituto de Investigación Biosanitaria ibs. Granada, University of Granada, 18071 Granada, Spain; Department of Bioprocessing & Bioanalytics, Fraunhofer Institute for Biomedical Engineering, 66280 Sulzbach, Germany; Institute of Human Genetics, Saarland University, 66421 Homburg, Germany; Center of Human and Molecular Biology, Saarland University, 66123 Saarbrücken, Germany; Chair for Clinical Bioinformatics, Saarland University, 66123 Saarbrücken, Germany; Center for Bioinformatics, Saarland Informatics Campus, Saarland University, 66123 Saarbrücken, Germany; Department of Bioprocessing & Bioanalytics, Fraunhofer Institute for Biomedical Engineering, 66280 Sulzbach, Germany; Institute of Human Genetics, Saarland University, 66421 Homburg, Germany; Chair for Clinical Bioinformatics, Saarland University, 66123 Saarbrücken, Germany; Center for Bioinformatics, Saarland Informatics Campus, Saarland University, 66123 Saarbrücken, Germany; Department of Neurology and Neurological Sciences, Stanford University School of Medicine, Stanford, CA, USA; Institute of Human Genetics, Saarland University, 66421 Homburg, Germany

## Abstract

MicroRNAs are regulators of gene expression. A wide-spread, yet not validated, assumption is that the targetome of miRNAs is non-randomly distributed across the transcriptome and that targets share functional pathways. We developed a computational and experimental strategy termed high-throughput miRNA interaction reporter assay (HiTmIR) to facilitate the validation of target pathways. First, targets and target pathways are predicted and prioritized by computational means to increase the specificity and positive predictive value. Second, the novel webtool miRTaH facilitates guided designs of reporter assay constructs at scale. Third, automated and standardized reporter assays are performed. We evaluated HiTmIR using miR-34a-5p, for which TNF- and TGFB-signaling, and Parkinson's Disease (PD)-related categories were identified and repeated the pipeline for miR-7-5p. HiTmIR validated 58.9% of the target genes for miR-34a-5p and 46.7% for miR-7-5p. We confirmed the targeting by measuring the endogenous protein levels of targets in a neuronal cell model. The standardized positive and negative targets are collected in the new miRATBase database, representing a resource for training, or benchmarking new target predictors. Applied to 88 target predictors with different confidence scores, TargetScan 7.2 and miRanda outperformed other tools. Our experiments demonstrate the efficiency of HiTmIR and provide evidence for an orchestrated miRNA-gene targeting.

## INTRODUCTION

MicroRNAs (miRNAs) are small non coding RNAs, which regulate the gene expression post-transcriptionally (1). Specifically, miRNAs repress protein translation of target mRNAs by binding to target sequences mainly in 3′ untranslated regions (3′UTRs) and less commonly in 5′ untranslated regions or open reading frames of their target mRNAs (2,3). Aberrant expression of miRNAs is not only a hallmark of various cancers and can be detected in tumor cells and body fluids including urine, saliva, and blood (4–6), but also in solid tissue, cerebrospinal fluid, and blood of neuropathological disorders like Alzheimer's Disease and Parkinson's Disease (PD) (7–10).

While miRNA gene targeting relies on a complementary binding of the seed region to the target gene, non-canonical binding between gene and miRNA also seems to have a deterministic influence on the targeting process (11,12). The limited understanding of the true complexity of the interactions between miRNAs and genes poses substantial challenges for the computational prediction of miRNA targets. In response to this challenge, many tools have been developed including TargetScan (13), PicTar (14), miRanda (15) and other consensus methods like miRWalk (16), which in turn combines the predictive power of several other predictors. The expectable number of targets per miRNA has not yet been reliably determined as a single miRNA can target between a few up to several hundred genes. Considering an overall search space of 62.5 million possible miRNA-gene interactions (25 000 human genes × 2500 human miRNAs) and the estimated number of targets of single miRNAs, a substantial class imbalance exists. Learning from imbalanced data however still poses challenges for machine learning in life sciences and beyond (17,18). When the *a priori* likelihood of a positive event gets small and the specificity is not close to an optimal value, the positive predictive value, i.e. the likelihood that a predicted event is actually positive, becomes extremely low (19).

Accumulating evidence suggests that the targetome of a miRNA is not randomly distributed across the transcriptome and that it covers genes of shared biochemical pathways. This information can support the design of prediction tools by increasing the specificity of target predictions while at the same time maintaining the sensitivity. Based on this assumption we previously developed the miRNA target pathway dictionary (20), which we subsequently extended into the miRPathDB (21), now existing in the second version (22). The wide-spread assumption that miRNAs target complex networks in an orchestrated manner to facilitate the discovery of new true positive targets has not yet been validated at scale. However, respective computational approaches, which use consensus prediction and target enrichment by pathways, motivate a systematic and standardized experimental validation of predicted targets. To validate miRNA targets, different experimental approaches exist with inherent advantages and disadvantages. One of the most common choices are reporter assays (23,24). As for the majority of similar technologies, limitations of reporter assays are known (25). In addition, manuscripts frequently report only one validated gene or small sets thereof. The miRTarBase in the most recent update 2020 (26) indicates that 6046 manuscripts describe 9679 human miRNA/gene pairs (including duplications) validated by reporter assays. Thus, on average, manuscripts validate only 1.6 targets. Additionally, 97% of the database entries are positive associations while negative results of reporter assays are frequently not reported.

**Figure 1.**
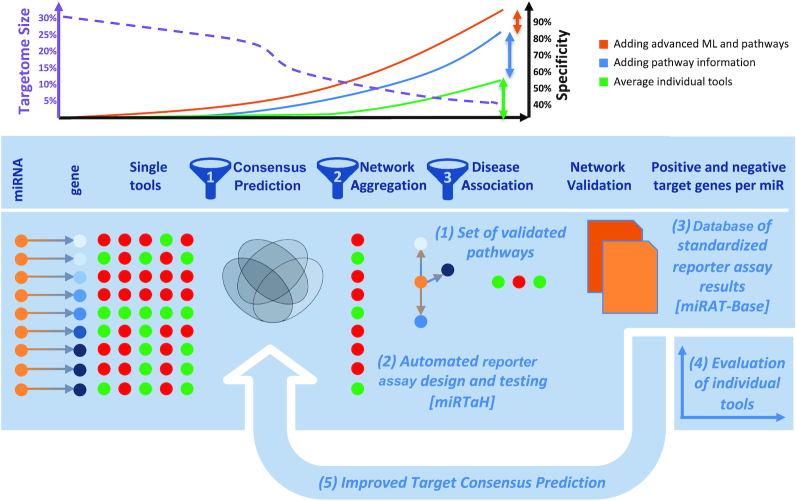
Study set-up, rational and contribution. The main goals of our study are to demonstrate an orchestrated targeting of miRNAs on specific pathways by experimental means and to provide novel useful resources for the scientific community. Originally, we increased the specificity of miRNA target interactions by in-silico approaches alone (green curve and green vertical arrow). By combining improved target selection strategies, we provide evidence for a higher specificity and validation rate in this study (blue curve and blue vertical arrow). We also provide evidence that in iterative improvements the specificity and validation rate can be further increased by improved target selection using advanced machine learning and pattern recognition techniques (orange curve and orange vertical arrow). Besides the main contribution of validated target pathways (1), our approach includes (2) a novel online tool miRTaH that facilitates reporter assay design at scale and (3) a database of validated pathways as well as positive and negative targets for single miRNAs. Finally, we demonstrate that a standardized target database is a valuable source for (4) evaluating the performance of individual tools and (5) improving target prediction and thus can support the development and evaluation of current and new miRNA target tools.

To address the challenge of identifying true miRNA targets in the overall search space of 62.5 million possible miRNA-mRNA interactions, we developed an approach termed **hi**gh-**t**hroughput **m**iRNA **i**nteraction **r**eporter assay (HiTmIR). Our approach combines computational and experimental work steps into a new pipeline. In the computational part, targets are first predicted by a consensus approach relying on well-established tools. Subsequently, targets are filtered by enriched pathways or diseases using the GeneTrail (27) pathway analysis software. From the enriched targetome a novel web-based software (miRTaH) can automatically design reporter sequences for luciferase reporter assays at scale, a task that is challenging and time consuming when performed manually. The final reporter assay target sequences can be obtained from various vendors and get handled by an automated microfluidic device. Therefore, our pipeline allows to identify a higher fraction of true miRNA target interactions than previously reported in an efficient manner. The identified targets and target pathways used to benchmark a variety of target prediction tools and databases in a low, medium, and high stringency set-up have been stored in the miRATBase data warehouse. The overall workflow of our study together with the main contributions to the field are shown in Figure 1.

We applied the HiTmIR workflow to two strongly conserved miRNAs, miR-34a-5p and miR-7-5p, which are both known to be deregulated in cellular PD models and brain tissue of PD patients (28–33). While miR-34a-5p is upregulated in PD, downregulation of miR-7-5p has been previously demonstrated to effect α-synuclein and to contribute to neurodegeneration (28,34). PD is the second most common neurodegenerative disorder following Alzheimer's Disease. Its prevalence strongly increases with age, resulting in 2% of the female world population and 7% of the male world population affected being over 85 years old (35). The clinical symptoms are caused by the loss of dopaminergic neurons within the *substantia nigra pars compacta* and coupled to the accumulation of α-synuclein into intraneuronal structures, known as Lewy bodies and Lewy neurites (36,37). In the last decade, the role of deregulated miRNAs in the pathogenesis of PD has been characterized, for example by the identification of several disease associated miRNAs involved in the progression of PD (38).

## MATERIALS AND METHODS

We here describe an overview of the applied methods and analyses. Further details on each of them are available in the supplement and online methods (Supplemental document).

### Automated dual luciferase reporter assay

For this assay 2–2.5 × 10^4^ HEK 293 T cells were seeded out per well of a 96-well plate (Eppendorf, Hamburg, Germany) by the liquid handling system epMotion 5075 (Eppendorf, Hamburg, Germany). HEK 293 T cells were transfected with 50 ng/well reporter vector with or without 3′UTR and 200 ng/well pSG5 empty vector or pSG5-miR-34a expression plasmid. Forty-eight hours after transfection cells were lysed and the cell lysates were prepared according to manual of the Dual-Luciferase^®^ Reporter Assay System (Promega, Madison, USA) and measured with the GlowMax navigator microplate luminometer (Promega, Madison, USA).

### miRNA expression plasmid and reporter constructs

The pSG5-miR-34a expression vector (Eurofins Genomics, Ebersberg, Germany) contains the nucleotides 9 151 617–9 151 816 of chromosome 1. The pSG5-miR-7 expression vector (Eurofins Genomics, Ebersberg, Germany) contains the nucleotides 88 611 724–88 612 046 of chromosome 15. For miR-34a-5p target gene validation, the sequences of the 191 3′UTRs of the TNF-, TGFB-signaling and the PD-related target genes were synthetized and the ∼490 nt long inserts were cloned into the pMIR-RNL-TK vector (Eurofins Genomics, Ebersberg). The 3′UTR sequences of *CREB1_1 mut, CREB1_2 mut, TNFSF14 mut, DNM1L_1 mut, DNM1L_2 mut, AKT2 mut, SMAD7 mut, BMP8B mut, SMAD2_1 mut, SMAD2_2 mut, TGFB2 mut* and *EP300 mut*, with mutated binding sites were synthetized and the inserts were cloned into the pMIR-RNL-TK vector. For miR-7-5p target validation, the sequences of the 160 3′UTRs of the PD-related target genes were synthetized and the ∼690 nt long inserts were cloned into the pMIR-RNL-TK vector (BGI, Shenzhen, China).

### Cell lines, tissue culture

Lund human mesencephalic (LUHMES) cells were purchased from the American Type Culture Collection (ATCC) and transfected for GFP-expression. The cells were cultured as previously described by Scholz et al. (39) in flasks pre-coated with 50 μg/ml poly-l-ornithin and 1 μg/ml Fibronectin. HEK 293T cells were cultured as described previously (40). SH-SY5Y cells were cultivated in DMEM (Life Technologies GmbH, Darmstadt, Germany) supplemented with 20% fetal bovine serum (Biochrom GmbH, Berlin, Germany), Penicillin (100 U/ml), and streptomycin (100 μg/ml). All cell lines were cultured for less than 3 months after receipt.

### Differentiation of LUHMES cells

For differentiation of LUHMES cells towards dopaminergic neurons, cells were cultured in advanced DMEM/F12 (Life Technologies GmbH, Darmstadt, Germany) supplemented with 1% N2-Supplement, 2 mM l-glutamine, 1 mM dibutyryl cAMP, 2 ng/ml GDNF and 1 μg/ml tetracycline. After 48 h, cells were trypsinized and seeded with 7.5 × 10^4^ cell/cm² in pre-coated flasks.

### Neurotoxin treatment and RNA isolation

To induce a PD-like phenotype, LUHMES cells were treated with 10 μM 1-methyl-4-phenylpyridinium (MPP+; Sigma Aldrich, Munich, Germany) 6 days after initiation of differentiation for 48 hours. Control cells were supplemented with H_2_O. For RNA-Isolation, cells were lysed by QIAzol Lysis Reagent (Qiagen, Hilden, Germany) and total RNA was isolated using the miRNeasy Mini Kit (Qiagen, Hilden, Germany).

### Immunocytochemistry

For immunocytochemistry staining of TH and D2R, LUHMES cells were cultured and seeded on pre-coated 8-well μ-slides (ibidi GmbH, Gräfelfing, Germany) with 7.5 × 10^4^ cells/cm^2^. Medium was exchanged 48 hours after re-seeding. The primary antibodies were diluted in PBS containing 1% bovine serum albumin and incubated at 4°C overnight. TH was stained using a polyclonal rabbit antibody (Cat# ab112, RRID: AB_297840, abcam, Cambridge, UK) and D2R was detected using a goat polyclonal antibody (Cat# ab32349, RRID: AB_2094849, abcam, Cambridge, UK). Images were taken with a Leica TCS SP8 microscope (Leica Microsystems, Wetzlar, Germany) and analyzed using LAS X software (version 3.5.5.19976, Leica Microsystems, Wetzlar, Germany).

### miRNA Microarray

miRNA expression profiles after MPP+ treatment in dopaminergic neurons were monitored by using Agilent miRNA Complete Labeling and Hyb Kit as well as Agilent SurePrint G3 Human miRNA 80 × 60K Microarrays (Cat. No. G4872A, miRBase release 21.0, Agilent Technologies, Santa Clara, CA, USA) as described previously (41). The raw microarray data has been deposited at the GEO database (GSE135151).

### Western blot

For western blot analysis of JNK3, SMAD2, SMAD7, CREB1, TH, CLOCK, PARK2 and GRIA4 4.5 × 10^5^ SH-SY5Y cells were seeded out per well of a six well plate. After 24 hours the cells were transfected either with the Allstars Negative Control (ANC) or with hsa-miR-34a-5p miScript miRNA Mimic (MIMAT0000255: 5′UGGCAGUGUCUUAGCUGGUUGU). For endogenous miR-34a-5p inhibition, cells were transfected with miScript Inhibitor Negative Control or anti-hsa-miR-34a-5p miScript miRNA Inhibitor (MIMAT0000255: 5 ′UGGCAGUGUCUUAGCUGGUUGU). Quantification of the western blots was carried out with Image Lab Software Version 5.2.1 (Bio-Rad Laboratories Inc., Hercules, CA, USA).

### Quantitative real-time PCR (qRT-PCR)

qRT-PCR was performed using miScript Primer Assay for hsa-miR-34a-5p, hsa-miR-7-5p, hsa-miR-181a-3p, hsa-miR-134-5p, hsa-miR-129-5p, hsa-miR-129-1-3p, hsa-miR-335-3p, hsa-miR-106b-3p, hsa-miR-412-5p, and Custom miScript Primer for hsa-miR-4284 (Qiagen, Hilden, Germany) and the StepOnePlus Real-Time PCR System (Applied Biosystems, Foster City, United States) following the manufacturer's protocol. RNU6B (Qiagen, Hilden, Germany) served as endogenous control. Statistical significance of differentially expressed miRNAs in MPP+ treated LUHMES as well as miR-34a-5p over-expression was analyzed by paired, two-tailed t-tests.

### Automated reporter assay construct generation using miRTaH

To facilitate the bioinformatics aided design of several hundred reporter assays we implemented miRTaH (miRNA Target assay Helper). In brief, miRTaH receives a paired list of miRNAs and genes as input query and searches for known miRNA-target interactions from public databases. Next, seed binding sites for each miRNA in the corresponding target gene 3′UTRs are searched. For a list of selected pairs, the 3′UTR sequences are displayed along with the detected miRNA binding sites and potential cut sites of restriction enzymes. Long sequences can be automatically split into any number of chunks, which then can be processed independently. Finally, the tool generates a report of the generated sequence inserts to be synthesized and cloned into reporter plasmids. As organisms, our web service supports *H. sapiens* and *M. musculus*. miRTaH is freely available online (https://www.ccb.uni-saarland.de/mirtah). Further descriptions on the tool are available from the supplemental materials.

### miRATBase—a database for validated targets and target pathways of miRNAs

To make the validated targets and target pathways accessible we implemented a data warehouse termed miRNA Reporter Assay Database (miRATBase). In this data warehouse we store for each miRNA the validated target pathways and the positive and negative target data sets. miRATBase is freely available online (https://www.ccb.uni-saarland.de/miratbase). In its current release, miRATBase contains over 500 target associations for four miRNAs. For each entry we also link to miRTarbase (26), miRBase (42), miRCarta (43) and MirGeneDB (44).

### MiRNA target prediction

Consensus lists of predicted miRNA targets were obtained using the online interface of miRWalk 2.0 (16). The prediction tools in addition to miRWalk comprise microT v4, miRanda, mirBridge, miRDB, miRMap, miRNAMap, PicTar2, PITA, RNA22, RNAhybrid and TargetScan (45–55). Target transcripts were sorted by the number of algorithms predicting a target and aggregated on the gene level for all entries surpassing the applied cut-offs. For TargetScan the version used during study conception and implementation (6.2) was benchmarked to the currently most recent version 7.2. To this end, all miRNA targets showing a conserved and a non-conserved target site were downloaded from the TargetScan website and processed in the same manner as the targets from version 6.2. Further, aggregated predictions have been extracted from the recent mirDIP release 4.1 (56). Specifically, we made use of 25 tools in the low, medium and high stringency set-up. The final list of evaluated tools thus comprises 88 (25 × 3 + 13) prediction tools with different stringencies.

### Statistical analysis

Analysis of microarray data was performed with GeneSpring (version 14.9, Agilent Technologies, Santa Clara, CA, USA). Statistical analysis of qRT-PCR and western blots was performed with Prism7.04 (GraphPad Software, La Jolla, USA) applying paired, two tailed t-tests. Quantification of the western blots was carried out with Image Lab Software Version 5.2.1 (Bio-Rad Laboratories Inc., Hercules, California, USA). Statistical analysis, including evaluation of the automated dual luciferase reporter assays, was performed with R version 3.6.3 applying two-tailed, one-sample t-tests. Heatmaps were generated using the pheatmap R package while all remaining plots were compiled with the ggplot2, cowplot and RColorBrewer packages. The association mining of predicted and validated targets was performed using the *apriori* function of the arules package. For data handling and transformations, the R packages tidyr, dplyr, stringr, data.table and openxlsx were utilized. To test the hypothesis whether 3′UTR lengths systematically influence the results we computed a ratio for each gene using the long and short assay RLUs and performed a one-sample, two-sided Student's *t*-test while setting μ equal to 1.

## RESULTS

### Overview on HiTmIR: a novel pipeline for validating target pathways of single miRNAs

**Figure 2.**
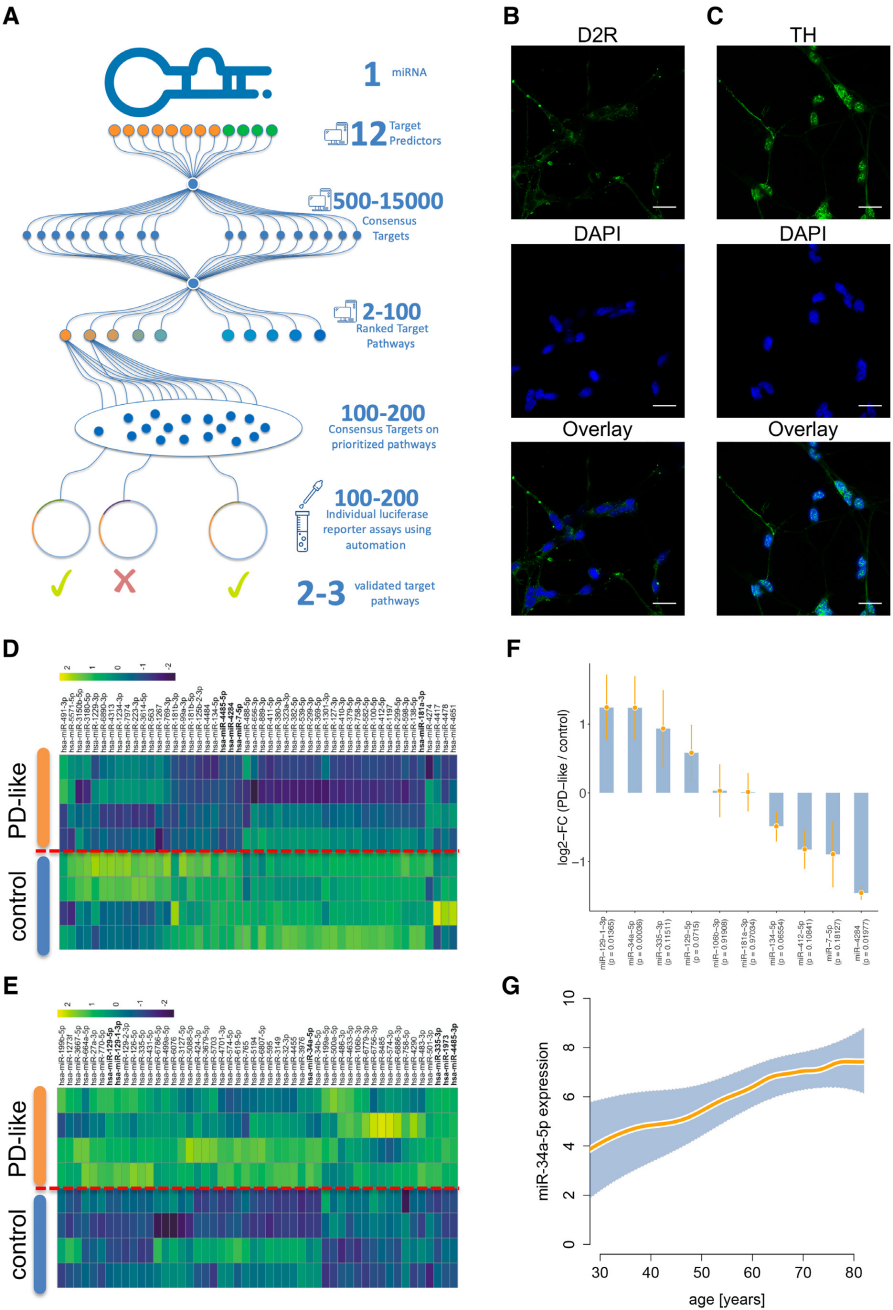
HiTmIR overview and representative selection of miR-34a. (**A**) Combined experimental and computational workflow of HiTmIR. Three computational steps are carried out consecutively before target gene sets are validated by an automated reporter assay. (**B**) Immunocytochemistry of D2R expression in differentiated LUHMES cells. (**C**) Immunocytochemistry of TH expression in differentiated LUHMES cells. (B, C) Expression of dopaminergic markers in differentiated LUHMES cells were analyzed by immunocytochemistry with antibodies against TH and D2R. The nuclei were visualized by DAPI staining. Scale bars are 25 μm. (**D**) Heatmap of the 50 most down-regulated miRNAs in LUHMES cells that were differentiated toward dopaminergic neurons and treated with MPP+ to induce a PD-like phenotype. (**E**) Heatmap of the 50 most up-regulated miRNAs. (D, E) Shown are z-scores of quantile-normalized expression values. (**F**) Validation of microarray results by qRT-PCR of up-regulated and down-regulated miRNAs. Bars present the log_2_ fold change between PD-like and controls together with the respective standard deviation. (**G**) Increased expression of miR-34a-5p in the blood of patients, spanning an age range from 20 to 80 years. The orange line shows a smoothed spline with 8 degrees of freedom and the shaded area represents the 95% confidence interval.

Our HiTmIR protocol, which was applied to two miRNAs, consists of three computational filters to increase the specificity of the target prediction and to reduce the size of the predicted targetome stepwise, followed by one experimental step (Figure 2A). The first computational filter includes a consensus target prediction (16). We then performed an over-representation analysis using GeneTrail2 (27) to identify enriched target pathways. Third, we added the disease association to the pathway information. Based on the significant categories, we built a consensus target gene set to narrow the experimental search space. A novel web-service supports the design of reporter constructs that are cloned into target plasmids and subjected to systematic experimental testing. To this end, a liquid handling system was programmed to perform an automated luciferase reporter assay in a 96-well format containing the commercially obtained constructs. This pipeline allows to detect and validate complete pathways for single miRNAs, which we exemplify for miR-34a-5p and miR-7-5p. The validated target pathways as well as the positive and negative targets are stored in a data warehouse, miRATBase, a resource for testing and evaluating new target prediction tools.

### Selecting microRNAs implicated in aging-related diseases to be screened with HiTmIR

To demonstrate the performance of HiTmIR we selected PD as role model. To further elucidate the role of miRNAs in PD, we differentiated lund human mesencephalic (LUHMES) cells to dopaminergic neurons and subsequently induced a PD-like phenotype using the neurotoxin MPP+ (1-methyl-4-phenylpyridinium). We verified the dopaminergic phenotype after differentiation by immunocytochemistry using tyrosine hydroxylase (TH) in combination with D2 receptor (D2R) as markers for dopaminergic neurons (Figure 2B and C). We analyzed four replicates each after stimulation with MPP+ and four according controls without MPP+ stimulation and identified 686 expressed miRNAs by genome-wide miRNA expression profiling. Following the stimulation by MPP+, we found 13 significantly deregulated miRNAs encompassing four down-regulated miRNAs including miR-7-5p and nine up-regulated miRNAs including miR-34a-5p (adjusted *t*-test *P*-values at an alpha level of 0.05) (Figure 2D and E). We validated the expression changes by qRT-PCR for 10 selected miRNAs comprising seven of the significantly deregulated miRNAs and three of the miRNAs with high fold-changes. The qRT-PCR analysis confirmed the deregulation for eight miRNAs including an up-regulation of miR-34a-5p and a down-regulation of miR-7-5p (Figure 2F, Supplemental Table S1). Since miR-34a-5p plays a crucial role in cancer and in neuropathologies, we investigated its abundance and dependency on age in blood of patients and controls. Analyzing a collection of 4393 individual blood samples (57), we examined miRNA expression of individuals who were between 30 and 80 years old (Figure 2G). We found a steady increase of miR-34a-5p expression over lifetime (*P* < 2.2 × 10^−16^). Since the observations suggest a prominent role of miR-34a-5p and miR-7-5p in neuropathological processes, these miRNAs were selected for systematic target pathway validation using the HiTmIR pipeline.

### Three computational filters decrease the predicted targetome size to 1% of the transcriptome

**Figure 3.**
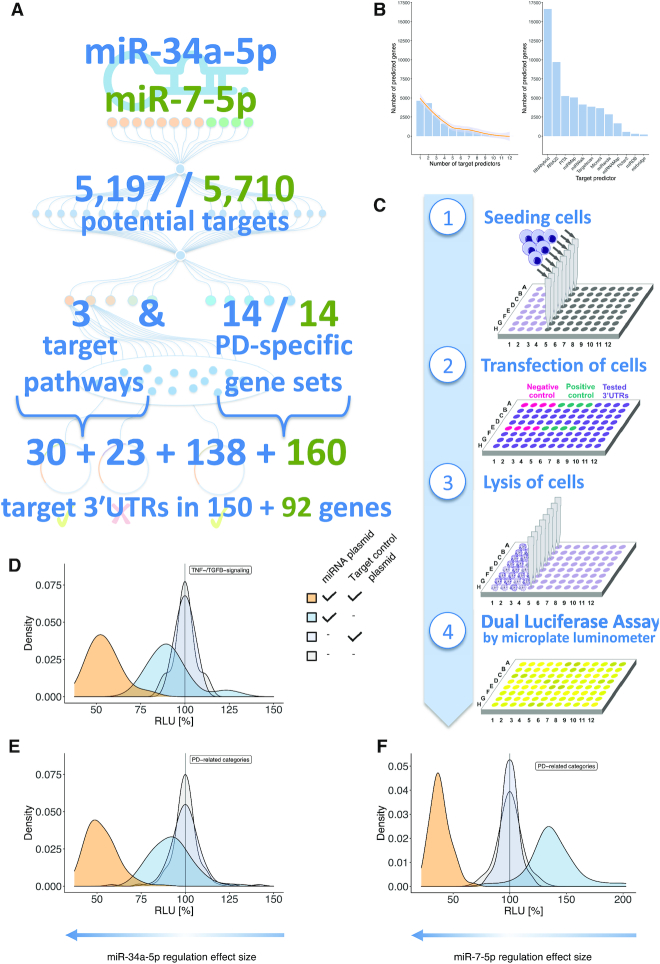
Application of HiTmIR to miR-34a-5p and miR-7–5p. (**A**) Adapted from the workflow in Figure 2A, the actual numbers of the application to miR-34a-5p (blue numbers) and miR-7-5p (green numbers) in the context of PD are shown. (**B**) Histogram of the number of predicted targets dependent on the number of tools predicting this target for miR-34a-5p. Most targets are predicted by one tool only. From the histogram, setting a threshold between three and five tools is a reasonable starting point because large parts of the unspecific hits are already excluded. We then set the initial number of predictions by requiring at least four tools to predict a target. The line represents a smoothed spline. The right-hand side plot of the panel displays the number of target predictions of the 12 individual tools. (**C**) The four experimental steps of the automated reporter assay required to validate target genes in a high-throughput manner. (**D**) Overview on HiTmIR results for miR-34a-5p in the TNF- and TGFB-signaling pathways. (**E**) Overview on HiTmIR results for miR-34a-5p in the PD-related categories. (**F**) Overview on HiTmIR results for miR-7-5p in the PD-related categories. (D–F) The x-axis displays the RLU while the y-axis depicts the density of experimental results. For each set, four curves of experimental transfection designs for targets of miR-34a-5p are shown; two times empty control plasmids (gray), empty miR plasmid + target control 3′UTR (light gray), miR-34a-5p plasmid + empty target control plasmid (blue), and the miR-34a-5p + target control 3′UTR plasmid (orange). The experimental transfection design for miR-7-5p was performed analogously.

The HiTmIR workflow was designed to start with a sensitive set of potential target genes, increasing the specificity in each of the computational steps (Figure 3A, Supplemental Table S2). One challenge in miRNA target prediction research are enormous sets of target genes for single miRNAs as exemplified for miR-34a-5p (Figure 3B). Seven of the 12 tools predict 20% or more of the transcriptome each. Considering the union of all target prediction algorithms basically the full transcriptome is identified as target for miR-34a-5p while each individual gene is only predicted by 2.4 of the 12 tools on average. The union of predictions thus represents a highly sensitive but very unspecific—and therefore unrealistic—representation of the targetome, calling for a more specific target set. While requiring more complex intersections, the number of targets predicted by a respective number of tools decreases significantly (Figure 3B). Around 75% of targets are already excluded by requiring an intersection of four tools to predict a gene, leaving 5198 target genes. At the same time, each of the genes is predicted on average by 5.2 tools. Still, this set is too unspecific and does likely not represent a reasonable targetome of miR-34a-5p. To add specificity, we next performed a pathway prediction as second filter step. By running an over-representation analysis in GeneTrail2 we detected a significant enrichment of target genes in 4507 pathways and biological processes (Supplemental Table S3). This analysis reduced the target gene set further by 33%. Yet again, the remaining number of 3475 genes likely represents an overestimation of the actual targetome. We then dissected targets enriched for pathways being pivotal for neurological diseases or for biological categories that have been associated with PD as a third filter. Specifically, we found 45 predicted miR-34a-5p target genes in the TNF-pathway and 32 in the TGFB-pathway, both of which have been studied in connection to neurological diseases (Supplemental Table S4). We further investigated categories relevant for PD. Here, GeneTrail2 highlighted a significant enrichment of 274 initially predicted miR-34a-5p targets in 14 PD categories, 10 of which are related to dopamine.

We compared the performance of the pipeline if applied to individual tools. For all 12 tools, we thus performed the exact same pathway analysis as for the consensus prediction (Supplemental Table S5). Here, we observed a higher concordance as compared to the gene-level prediction. On average, the pathways were predicted by 8.2 tools while using the above sketched consensus approach only 5.2 tools predicted a gene (*P* < 10^−5^). While most of the more complex KEGG pathways were covered by basically all tools (Dopaminergic synapse by all tools, TNF signaling pathway and TGF-beta signaling pathway by 11 tools), some of the smaller yet important Gene Ontology biological processes would have been missed by individual tools (Dopamine metabolism (six tools), Pink/Parkin Mediated Mitophagy (four tools) or dopamine catabolic process (three tools)). These results suggest that incorporating the information of different tools can add to the identification of relevant pathways, especially if these pathways are small.

To identify novel miR-34a-5p targets we relied on the information from the original consensus prediction but excluded all predicted target genes that did not have canonical binding sites and those targets, which were already validated by others according to the miRTarBase (58). Thereby, we obtained a final set of 150 target genes. For some of the predicted target genes, sequence analysis revealed multiple miRNA binding sites within the 3′UTR. To cover longer 3′UTRs that harbor multiple target sites, we split the sequence stretches into different segments to allow for testing of the miRNA effect on each target site separately (Supplemental Table S6). To this end, 3′UTR segments were cloned and separately tested. The respective segments were numbered consecutively starting at the 5′ end, with the number of the corresponding segment added to the plasmid name (as for example pMIR-CLOCK_1 and pMIR-CLOCK_2). In sum, we cloned 30 predicted target 3′UTRs for the TNF-pathway, 23 for the TGF-beta-pathway and 138 for genes associated with PD pathways. In generating the reporter assay constructs (cf. Supplemental Table S6) we recognized the need for a tool that automates this step and implemented the miRNA target assay helper tool miRTaH. The tool, which is freely available as web service (https://www.ccb.uni-saarland.de/mirtah), generates reporter construct sequences for arbitrary miRNA gene target pairs for *H. sapiens* and *M. musculus*. miRTaH supports binding site matching, restriction enzyme site analyses, and selection as well as modification of target sequences. The final sequences can be stored, exchanged, and downloaded easily.

We repeated the above described computational strategy for miR-7-5p. The consensus prediction yielded 5710 unique target genes (Supplemental Table S7). The analogous over-representation analysis returned 4484 pathways and functional categories (Supplemental Table S8). Since miR-7-5p is well described in the context of PD by targeting α-synuclein (34), we focused on the predicted targets for the same set of PD-related categories as screened for miR-34a-5p (Supplemental Table S9). Following the filtering with the same criteria, we generated reporter construct sequences and split 3′UTRs accordingly to a different size of ∼700 nts (Supplemental Table S10). Altogether, 150 and 92 genes were tested by automated dual luciferase assays for miR-34a-5p and miR-7-5-p, respectively.

### HiTmIR performance is comparable to manual reporter assays

We tested all 351 selected target gene 3′UTRs using the experimental part of HiTmIR (Figure 3C). To control the validity of the assay, each 96-well plate contained two positive controls in variable wells to exclude positioning-effects. The miR-34a-5p positive controls of the TNF/TGFB-signaling assays showed similar RLU distributions to those of the PD-related categories (Figure 3D and E, Supplemental Table S11). Upon co-transfection with miR-34a-5p, the positive control pMIR-TCRA showed a significant down regulation of the relative luciferase activity (relative light units; RLU) to 54.7% for TNF/TGFB-assays (*P* ≤ 0.001) and to 52.5% for PD related assays (*P* ≤ 0.001), comparable to previous effects obtained by manual assays (59). Next, we repeated the experiments for miR-7-5p. Following co-transfection of miRNA and target plasmid we also found a clear downshift of the RLU values to a mean of 38.6% (Figure 3F, Supplemental Table S12).

### HiTmIR validates 40% of miR-34a-5p targets in TNF- /TGFB-signaling pathways

**Figure 4.**
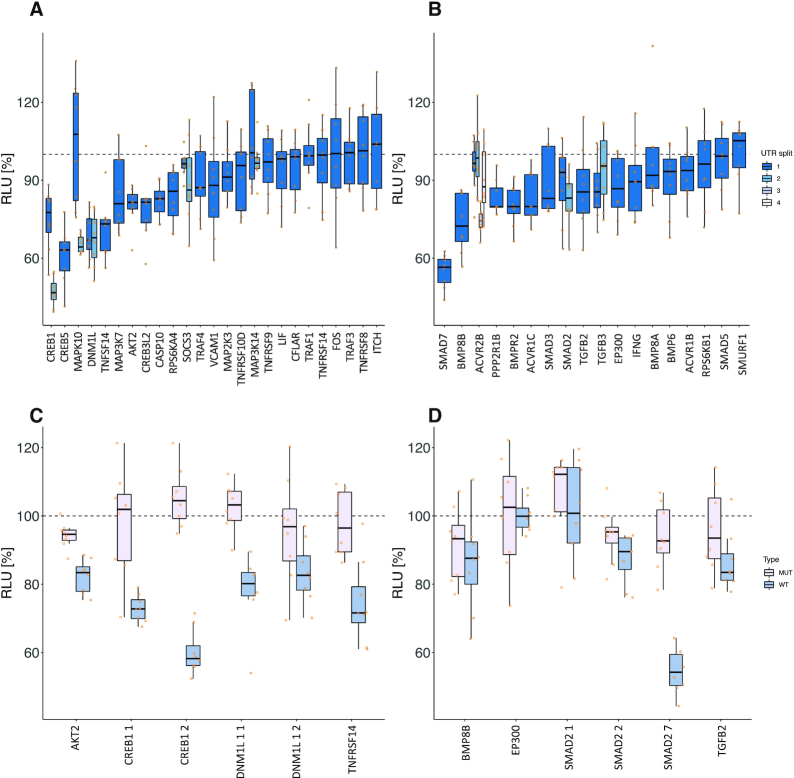
Detailed experimental results of HiTmIR for miR-34a-5p in TNF- and TGFB-signaling. (**A**, **B**) RLU values for eight replicates for each 3′UTR from selected target genes. The dashed line shows the normalized reference level, i.e. the expected level with no effect. (A) Results for pre-selected genes from TNF-signaling. (B) Results for pre-selected genes from TGFB-signaling. (**C**, **D**) RLU values for eight replicates for each wild-type and mutated (binding-site knock-out) 3′UTR from selected target genes. The dashed line shows the normalized reference level, i.e. the expected level with no effect. (C) HiTmIR results for binding-site knockout mutants of selected genes from TNF-signaling pathway. (D) HiTmIR results for binding-site knockout mutants of selected genes from TGFB-signaling pathway.

Out of the 30 tested 3′UTR sequences of the TNF-signaling pathway, 12 (40%) reporter constructs showed a significant RLU down regulation upon co-transfection with miR-34a-5p (Figure 4A, Supplemental Table S13). For TGFB-signaling, 9 of 23 (39%) tested target 3′UTRs showed a significant RLU reduction (Figure 4B). To verify the direct binding of miR-34a-5p to its predicted target sites, we mutated the binding sites and performed comparative HiTmIR experiments between the wild type constructs and the mutated reporter vectors (Figure 4C and D, Supplemental Table S14). For each signaling pathway, we chose six positively tested target gene segments. In sum, we tested CREB1_1, CREB1_2, TNFRSF14, DNM1L_1, DNM1L_2 and AKT2 from TNF-signaling, and SMAD7, BMP8B, TGFB2, SMAD2_1, SMAD2_2 and EP300 from TGFB-signaling. We verified the binding of miR-34a-5p to its predicted target sites for six 3′UTRs showing a significant difference in RLU after mutation. For the non-significant cases, the assay results still suggested a trend to lower RLU values upon a knockout of binding sites.

### HiTmIR validates 60% of PD-related pathways for miR-34a-5p and miR-7-5p

**Figure 5.**
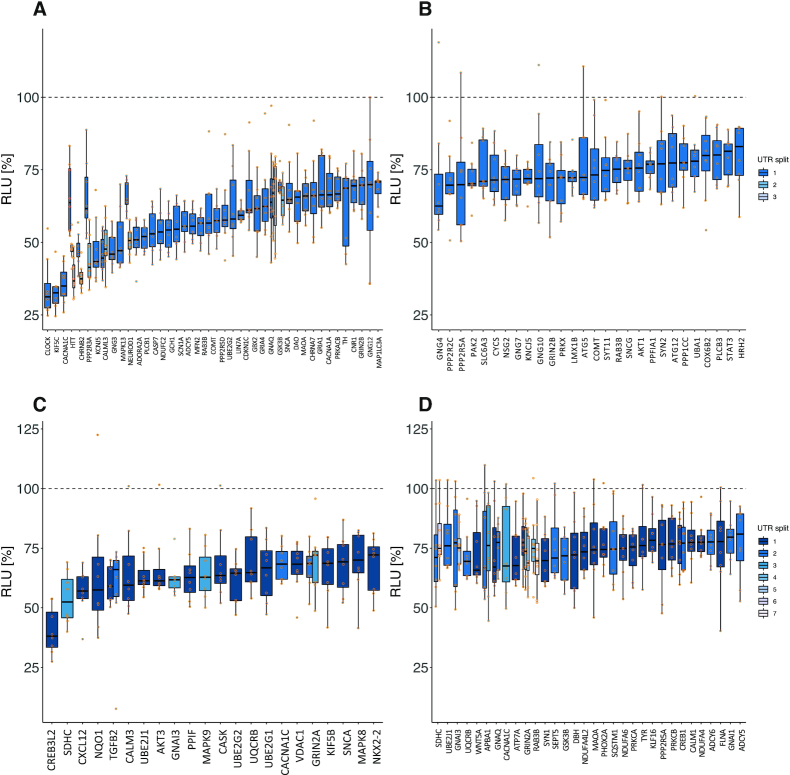
Detailed experimental results of HiTmIR for miR-34a-5p and miR-7–5p in PD-related categories. (A–D) RLU values for eight replicates for each 3′UTR from selected target genes. The dashed line shows the normalized reference level, i.e. the expected level with no effect. (**A**) Results for miR-34a-5p in the PD-related gene sets. Shown are the genes for which mean RLU was less or equal than 70%. (**B**) Analogous to (A) but with mean RLU between 70% and 80%. (**C**) Results for miR-7-5p in the PD-related gene sets. Shown are the genes for which mean RLU was less or equal than 70%. (**D**) Analogous to (C) but with mean RLU between 70% and 80%.

We applied the experimental pipeline of HiTmIR to the predicted and PD-related 3′UTR target genes of miR-34a-p and miR-7-5p (Supplemental Tables S13 and S15). Upon co-transfection with miR-34a, we detected a significant reduction (*P* < 0.05) of the RLU for 119 target 3′UTRs predicted by at least one algorithm (86.2%). Grouping the plasmids into RLU ranges, we found 51 cases in the range between 33% (KIF5C) and 70% (GSK3B_1) (Figure 5A). We observed a less pronounced decrease between 70% and 80% for 28 target 3′UTRs (Figure 5B). We next evaluated how the cut-off for the minimal number of consensus predictions potentially influences the results. Employing the cut-off, which we already used in the TNF-/TGFB-signaling validation, we observed a slight drop of the validation rate to 84.4%. However, only 39 (32.8%) genes that were predicted by at least four algorithms were removed due to non-detectable binding sites as compared to the 235 (68.7%) genes that were predicted by at least one algorithm. These results suggest an inflated false-positive rate for the genes predicted by a small number of tools only.

Of the 160 sequences tested for miR-7-5p, 106 (66.3%) were significant (*P* < 0.05). Mapping the constructs into the ranges of mean RLUs we only observed 24 targets under 70% (Figure 5C) and 40 targets (Figure 5D) of moderate reduction. These results suggest the validation rate of HiTmIR to primarily depend on the chosen cut-offs as well as the miRNAs under investigation. To elaborate on the relation between high validation rates and the chosen cut-off (standard) parameters per miRNA, we enumerated a set of thresholds for both the minimum mean RLU and the minimum *P*-value cut-offs and computed the corresponding validation rates (Supplemental Table S16). We found that even with permissive cut-offs (*P* < 0.005 & mean RLU < 80%) the validation rates for the PD-related target sets of miR-34a-5p and miR-7-5p remained competitive with 55% and 35%, respectively. After showing a significant decrease of target expression upon miRNA transfection, we next asked whether the protein expression levels are decreased accordingly.

### miR-34a-5p effects target protein expression in SH-SY5Y cells

**Figure 6.**
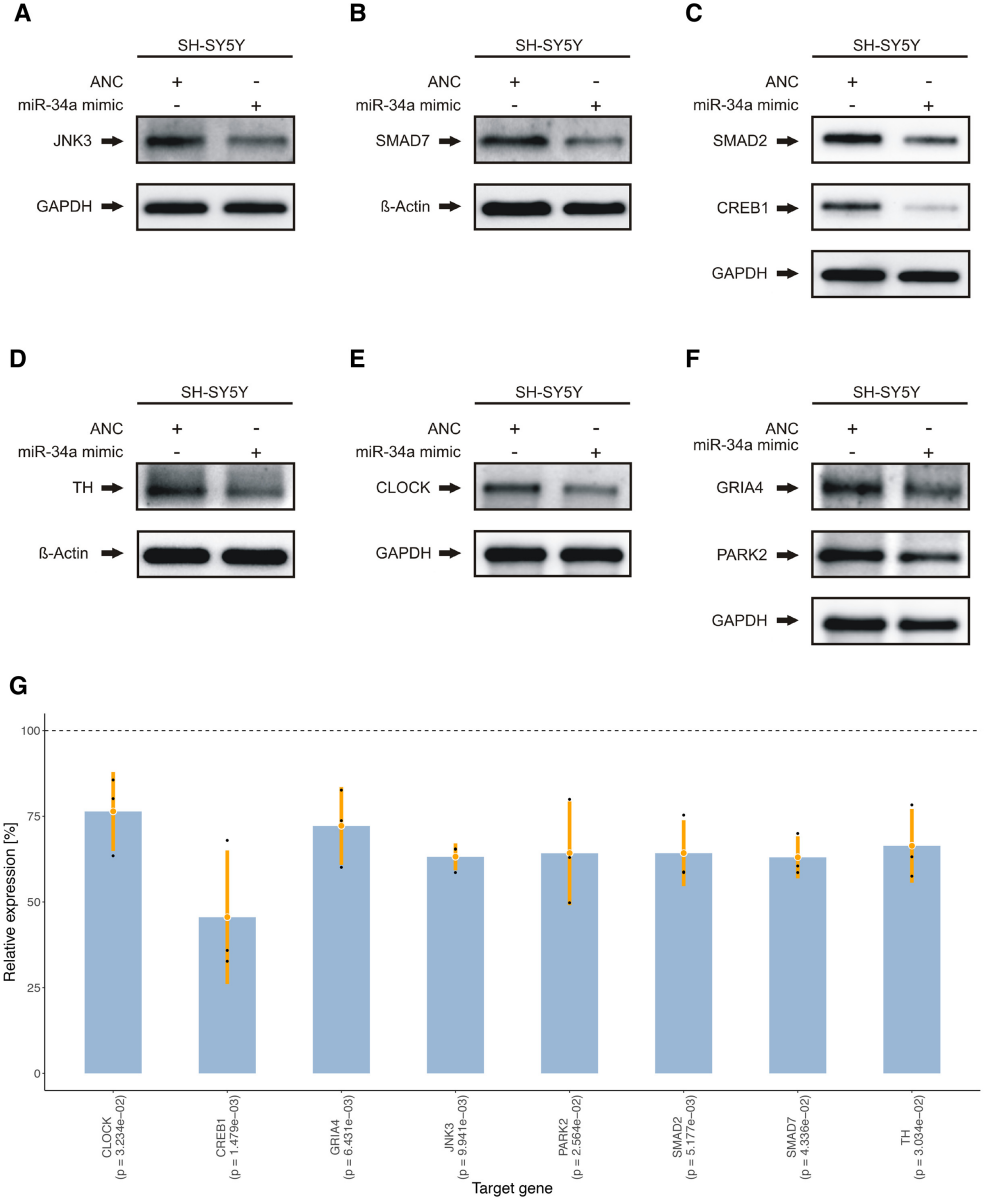
Western blot analysis of JNK3, SMAD7, SMAD2, CREB1, TH, CLOCK, PARK2 and GRIA4 in miR-34a-5p over-expressing cells. SH-SY5Y cells were transfected either with ANC or miR-34a-5p mimic. Forty-eight hours after transfection, the endogenous protein levels were analyzed by western blotting using specific antibodies against the aforementioned proteins. GAPDH or β-Actin served as loading control. One representative western blot out of three independent experiments is shown, respectively. All three western blots were quantified by densitometry using the Image Lab Software. (**A**) Western blot results for JNK3. (**B**) Western blot results for SMAD7. (**C**) Western blot results for SMAD2 and CREB1. (**D**) Western blot results for TH. (**E**) Western blot results for CLOCK. (**F**) Western blot results for GRIA4 and PARK2. (**G**) Combined expression analysis for genes from (A) to (F) tested by western blot analysis. The y-axis displays the relative expression levels with respect to the ANC (100%, dashed line). Each blue bar represents the triplicates (black dots) of a gene with mean (orange dot) and a range of two times the standard deviation (orange lines). *P*-values shown in parenthesis were computed using two-tailored, paired Student's *t*-tests.

**Figure 7.**
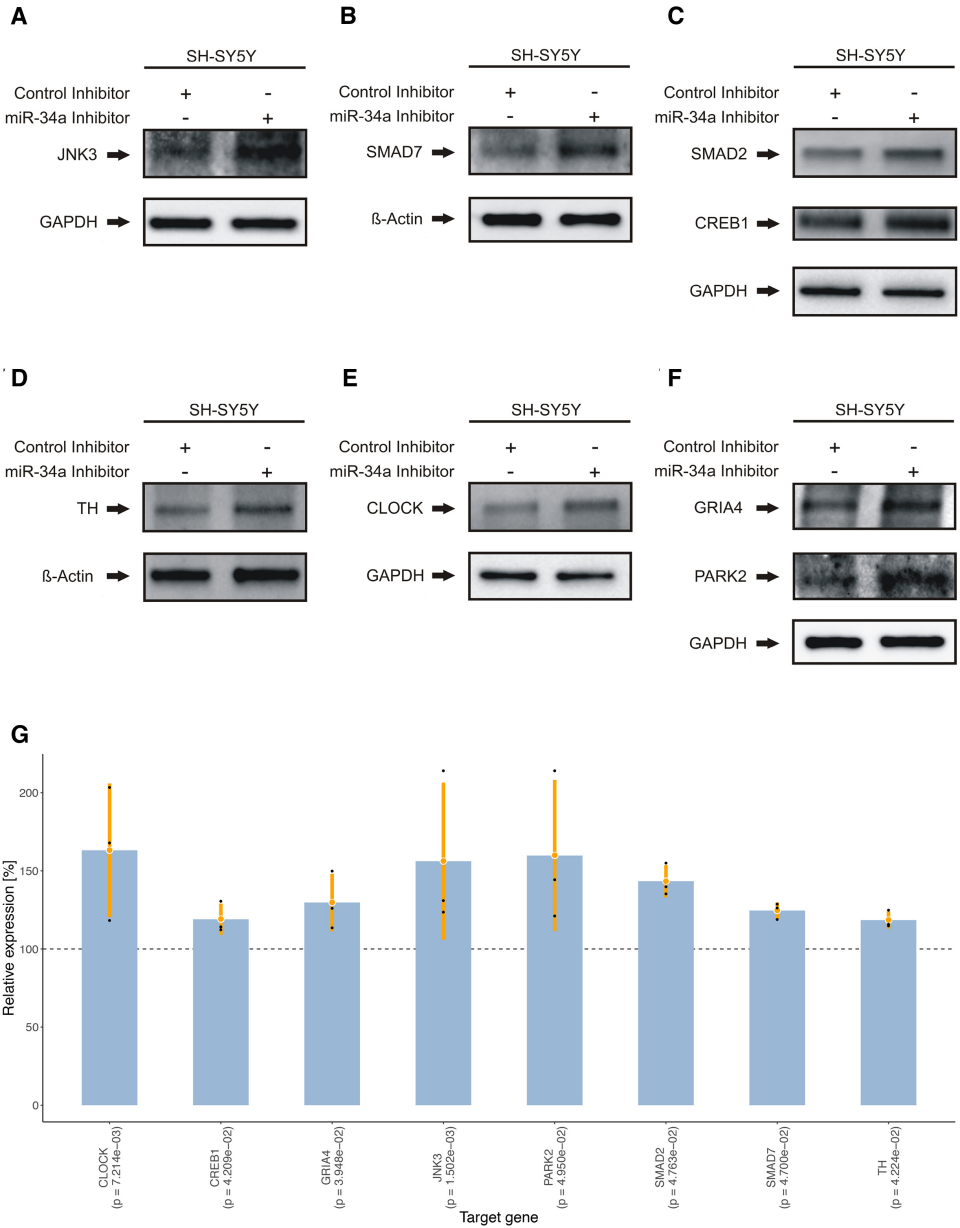
Western blot analysis of JNK3, SMAD7, SMAD2, CREB1, TH, CLOCK, PARK2 and GRIA4 in miR-34a-5p inhibitor transfected cells. SH-SY5Y cells were transfected either with inhibitor control or miR-34a-5p inhibitor. Forty-eight hours after transfection, the endogenous protein levels were analyzed by western blotting using specific antibodies against the aforementioned proteins. GAPDH or β-Actin served as loading control. One representative western blot out of three independent experiments is shown, respectively. All three western blots were quantified by densitometry using the Image Lab Software. (**A**) Western blot results for JNK3. (**B**) Western blot results for SMAD7. (**C**) Western blot results for SMAD2 and CREB1. (**D**) Western blot results for TH. (**E**) Western blot results for CLOCK. (**F**) Western blot results for GRIA4 and PARK2. (**G**) Combined expression analysis for genes from (A) to (F) tested by western blot analysis. The y-axis displays the relative expression levels with respect to the control inhibitor (100%, dashed line). Each blue bar represents the triplicates (black dots) of a gene with mean (orange dot) and a range of two times the standard deviation (orange lines). *P*-values shown in parenthesis were computed using two-tailored, paired Student's *t*-tests.

To investigate the effects of miR-34a-5p targeting on the endogenous protein levels, SH-SY5Y cells were transfected by miR-34a-5p mimics or by ANC as a non-targeting control. We confirmed the over-expression of miR-34a-5p in the transfected SH-SY5Y cells by qRT-PCR (Supplemental Table S17). We next analyzed the endogenous protein levels of JNK3, SMAD7, SMAD2, CREB1, TH, CLOCK, GRIA4 and PARK2 each in three independent experiments by western blotting using specific antibodies (Supplemental Table S18). We observed significantly reduced endogenous protein levels for all tested proteins (Figure 6A–F) ranging from 46% for CREB1 (0.001 ≤ *P*-value ≤ 0.01) to 76% for CLOCK (*P*-value ≤ 0.05) (Figure 6G). To further validate miR-34a-5p endogenous targeting, we transfected SH-SY5Y cells with miR-34a-5p inhibitor or an inhibitor control and analyzed the endogenous protein levels of JNK3, SMAD7, SMAD2, CREB1, TH, CLOCK, GRIA4 and PARK2 each in three independent experiments (Supplemental Table S19). In line with the previous observations, we found significantly induced endogenous protein levels for all of the tested proteins ranging from 118% for TH (*P*-value ≤ 0.05) to 163% for CLOCK (0.001 ≤ *P*-value ≤ 0.01) (Figure 7).

### Variation in cloned 3′UTR lengths does not lead to a systematic bias

Since the validation rates of HiTmIR varied between miR-34a-5p and miR-7-5p, we asked whether this is confounded by the fact that 3′UTR splits of varying lengths were transfected. As independent control experiments we selected nine target 3′UTRs of miR-34a-5p and created reporter constructs containing the full-length 3′UTR sequence. The full-length 3′UTR sequences (∼991 nts) were approximately two times the length of the shorter sequence chunks (∼477 nts) (Supplemental Table S20). Although several cases could be identified where the shorter 3′UTR sequence showed either a better or worse mean RLU, these differences were not significant on the overall distribution (*P* = 0.9962, cf. Materials and Methods). As a conclusion, the length of the 3′UTR reporter constructs does not significantly skew the distribution of RLU values obtained, as long as the technically upper limit (∼1500 nts) is not surpassed.

### Evaluating the performance of single tools toward a more accurate consensus prediction

**Figure 8.**
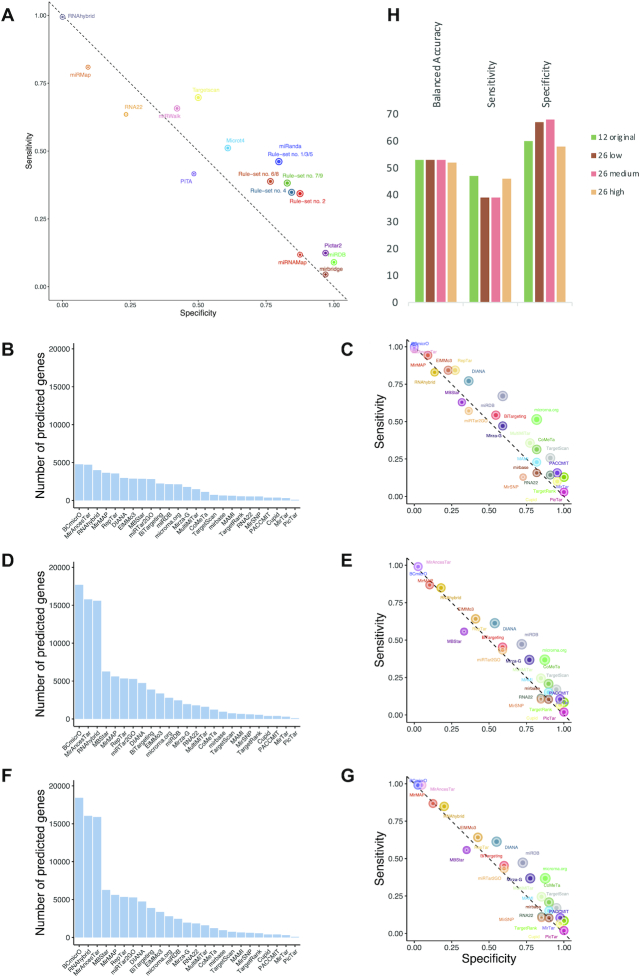
Performance evaluation of individual tools and association rules. (**A**) The scatter plot shows the specificity and sensitivity of the 12 individual tools and the association rules. The point size of the tools and rules correspond to the balanced accuracy. (**B**) Targetome sizes for the most stringent parameters for the new set of tools. (**C**) Specificity and sensitivity of the most stringent parameter set for the new set of tools. (**D**) Targetome sizes for the medium stringent parameters for the new set of tools. (**E**) Specificity and sensitivity of the medium stringent parameter set for the new set of tools. (**F**) Targetome sizes for the least stringent parameters for the new set of tools. (**G**) Specificity and sensitivity of the least stringent parameter set for the new set of tools. (**H**) Balanced accuracy, specificity, and sensitivity for the four tool groups presented in A, C, E, G.

By design, the HiTmIR system facilitates validation of miRNA targets that are predicted and prioritized by *in silico* methods. In turn, it does not only provide a set of validated target pathways but also positive and negative sets of targets for miRNAs. These can be used to evaluate the performance of individual target predictors, utilized to test new individual tools, or used to evaluate consensus prediction. First, we calculated the performance of the individual tools that were originally contained in the target gene selection step to determine whether and how performance varies between the tools (Figure 8A). Our results suggest one set of tools (mirbridge, miRDB, miRNAMap and Pictar2) to be very specific. While this specificity is on a level we are seeking for, it here comes at the price of a sensitivity of only 9%. On the other extreme, RNAhybrid shows a sensitivity of 99.4% but also zero specicitity on our data set. As previously suggested, TargetScan (6.2) and miRanda show a well-balanced specificity and sensitivity. The only other tool that performs similarly well is MicroT v4. However, it is in the nature of successful tools that they are constantly improved. Therefore, we evaluated more recent programs (56). Altogether, 25 tools were tested and most notably for these tools low (Figure 8B and C), medium (Figure 8D and E) and high (Figure 8F and G) confidence sets of targets were acquired to evaluate the performance. Additionally, we included the 12 original tools and TargetScan 7.2. In total we evaluated 88 tools at varying levels of prediction stringency. For each of the tools, we computed the specificity, sensitivity, balanced accuracy, and other measures such as precision, recall, and the F1 score (Supplemental Table S21). As expected, the number of predicted targets generally decreases with stringency increasing. Still, the most stringent sets yield targetome sizes over 20% of the transcriptome. The high confidence set retained a sensitivity, specificity and balanced accuracy of 47%, 60% and 53%. The medium confidence set 39%, 67% and 53%, respectively. The low confidence set yielded 39%, 68% and 53%, almost identical to the medium confidence set. Most importantly, the original set we used reached 46%, 58% and 52% sensitivity, specificity and balanced accuracy, similar to the high confidence set of mirDIP (Figure 8H). The most remarkable difference between the four groups of tools was the increased sensitivity of the high confidence sets, at the cost of the lowest specificity. Of note, there was no tool that clearly outperformed all others, i.e. reaching exceptional specificity and sensitivity. The best-balanced accuracies, exceeding values of 60%, were reached for microrna.org, miRDB, miRanda and TargetScan (7.2).

We then evaluated how an updated algorithm improved the results on the example of TargetScan and compared version 6.2 (the available version when we originally implemented HiTmIR) with the most recent version 7.2. We specifically asked whether a tool update has an impact on single target genes and on the validation success rate. With respect to the original gene sets we observed an overlap of 3384 target genes, for which the newer version had an additional 1000 targets while 444 former targets were not predicted anymore. Most intriguingly, the pathway prediction was 100% concordant between TargetScan 6.2 and TargetScan 7.2 (Supplemental Table S5). In predicting more targets, we might expect also an increased false positive rate but for the genes involved in our study we observed three more true positives and two more true negative genes. For TargetScan 6.2 we computed 124 TP, 32 TN, 32 FP and 54 FN. For TargetScan 7.2 the numbers slightly changed to 126 TP (+2), 33 TN (+1), 31 FP (–1) and 52 FN (–2). The balanced accuracy improved from version 6.2 (59.8%) to 7.2 (61.2%) by 1.4% and in a non-significant manner (*P* > 0.05). Although the overall improvement is statistically not significant, the data nonetheless indicate that advancing individual target tools can improve the accuracy further. The varying performance of the single tools and limitations in consensus approaches as applied in our study also motivates the question whether the obtained wet-lab results in turn can be used to rank the prediction tools used in the first step. To this end, we concatenated the predictions of the 12 tools for miR-34a-5p and miR-7-5p to create a binary matrix. Next, we filtered for the combination of miRNA and validated targets and added a binary response vector (1 = validated, 0 = not validated) using the standard cut-off (*P* < 0.05) on the experimental HiTmIR results. Based on an association mining procedure, we searched for a set of rules with high confidence to indicate tools or combinations of such, which are most informative towards the outcome vector. After setting a stringent cut-off for the confidence (≥80%) and a moderate level for the minimal support (≥25%), we computed nine rules (sets of tools) that could help to improve the validation rate in a retrospective manner (Supplemental Table S22). For example, the rule to combine the predictions of miRanda and TargetScan has the largest effect on the validation rate. These results suggest that several combinations of the tools incorporated in our pipeline give a better consensus prediction. Also, this means that the likelihood of a validation to turn out positively is higher than for any other single tool or combination of such. By contrast, negating the binary values of the outcome vector and repeating the association analysis did not yield any signature with high confidence (≥0.4) or support (≥0.3). This shows that non-validated targets are not predicted systematically by any subset of tools. We recommend to potential HiTmIR users to compare the global consensus prediction with the predictions obtained from the derived signatures of tools.

## DISCUSSION

With millions of theoretically possible interactions between miRNAs and mRNAs the known human miRNA targetome is far from being complete. Thus, novel methods combining high-throughput experimental and computational methods are in great demand to bring the field closer towards a comprehensive characterization of the targeting mechanisms of miRNAs. Although >100 prediction tools have been proposed, performance largely varies and even well performing tools typically report between several hundred and many thousand targets per miRNA (60). In the light of an expected low *a priori* likelihood of a miRNA targeting a gene, the specificity is of crucial importance. Considering a scenario with a low *a priori* likelihood and a specificity below 80%, the positive predictive values gets extremely low. To partially address this issue, consensus predictions of multiple predictors were used to further sharpen the set of predicted genes. Nonetheless, the methodological similarity of the approaches and their feature sets certainly influence the effectiveness of this filtering technique, still leading to high number of potential target candidates. Researchers face the situation to validate either a small set of selected candidates using traditional low-throughput techniques like reporter assays or to perform unbiased genome-wide assays that exhibit high levels of noise and complicate down-stream analysis. In addition, recent findings suggest that miRNAs orchestrate entire target pathways, an observation that has been claimed repeatedly, but never systematically been shown (59,61).

Therefore, we developed the novel HiTmIR pipeline, specifically designed to close the gap by mapping predicted targets to enriched pathways. The pipeline allows to rapidly design hundreds of recombinants based on 3′UTR sequences, which are tested using an automated parallel dual luciferase assay system. Our requirements for targets to be predicted by at least four tools followed by the filtering of enriched pathways or gene sets, improves state-of-the-art validation rates.

As for the experimental arm of our strategy, we implemented an automated dual luciferase reporter assay for high-throughput miRNA target gene validation. Although luciferase-based target validation has its inherent limitations, reporter assays provide an important piece of evidence whether a miRNA directly binds to its predicted mRNA target site. Here, we addressed two major limitations of reporter assays. First, cloned target sequences mostly do not represent the entire sequence context of the target site. Second, miRNAs are over-expressed in a non-physiological context (62). Examining the effects of different 3′UTR length on the results of reporter assays, we detected altered RLUs for varying 3′UTR lengths but no systematic bias that significantly influences the overall results. Moreover, we confirmed physiological targeting by miRNA inhibition. Using western blotting on transfected cells, we confirmed miRNA targeting for all of the proteins that were indicated as miR-34a-5p targets by reporter assays. To date, there is no gold-standard method for defining target gene regulation by miRNAs. Other high-throughput approaches like the combination of immunoprecipitation of argonaute (AGO) family members with next-generation sequencing (AGO-HITS-CLIP) do only provide evidence of miRNA-mRNA interaction but do not reflect the functional consequences (63). Comparable, high-throughput approaches that are also based on dual luciferase assays reported a significantly lower conformation rate for positive miRNA–mRNA-interactions (63,64). HiTmIR combines the computational target prediction, pathway analysis, automated reporter construct design as well as automated dual luciferase reporter assay for the identification of miRNA targets within a cellular signaling pathway and yields improved target validation rates.

To demonstrate the performance of HiTmIR we selected miR-34a-5p and miR-7-5p as use cases in the context of PD-related pathways. Besides specific evidence for an altered miRNA expression associated with PD, there is a systemic increase of miR-34a-5p with age correlating with the prevalence of neurodegenerative diseases along the lifespan. Also, the observed down-regulation of miR-7-5p has been previously described to effect α-synuclein and to contribute to neurodegeneration (34). Also in a MPTP induced PD model in mice, this miRNA was reduced (33). For both miRNAs, we showed up-scaled reporter assays to resemble the performance of manually performed experiments. Furthermore, automation allows to test batches of targets under replicable conditions. For TNF- and TGFB-signaling selected from our computational workflow, HiTmIR validated about 40% of target genes for miR-34a-5p. Validation rates were further improved for the PD-related categories, with a mean validation rate of 60% when considering both miRNAs. Moreover, we independently validated many of the targets for miR-34a-5p using binding site knockout assays and western blots with miRNA mimics and inhibitors. We then elaborated to which extent the performance depends on several parameters in the pipeline and argued that it can be miRNA specific. For the sake of simplicity, we calculated the validation rate primarily on a per 3′UTR basis as there is no gold-standard to compute it per gene. According to a technical limitation of reporter assays, several 3′UTRs had to be split into smaller constructs, an auxiliary technique that seems not to cause a systematic bias on the validation rates. Thereby, several justifiable ways exist to aggregate the HiTmIR results to compute a validation rate on the gene-level. For example, a simple rule could be to classify a gene as validated if at least one 3′UTR sequence of that gene is regulated by the chosen miRNA. Using the proposed stringent cut-offs (*P* < 0.005 & mean RLU < 80%) in combination with this rule yields a validation rate of 58.9% for miR-34a-5p and 46.7% for miR-7-5p on the gene-level for the PD-related pathways.

Our computational analysis highlighted TNF- and TGFB-pathways as target sets for miR-34a-5p and further 14 PD-related categories for miR-34a-5p and miR-7-5p. Regulation of different target genes by these miRNAs in the context of PD has been described only for a limited number of genes (30,34,65). Applying our new computational and experimental strategy HiTmIR, we demonstrate a complex regulation of cellular pathways for both miRNAs. This has been broadly claimed, but has never been proven to such an extent, especially in a disease-specific context. Via multiple points of interaction, deregulation of these miRNAs strongly impacts the signaling pathways and likely promotes cell death of dopaminergic neurons. As for example, TNF-signaling and TGFB-signaling regulate crucial processes in the central nervous system including synapse formation, synapse regulation, neurogenesis, regeneration and general maintenance of neuronal cells (66–69). Thus, a reduced TGFB-signaling by miR-34a-5p could promote nigrostriatal degeneration (68). Beyond this, we identified not only several PD-associated target genes for miR-34a-5p and miR-7-5p but also multiple targets that are crucial for dopamine metabolism and signaling. In this context, we identified the tyrosine hydroxylase, which converts l-tyrosine to l-dihydroxyphenylalanine (l-DOPA) and is a key enzyme of the dopamine metabolism as direct target of miR-34a-5p. Loss of TH is found within the striatum in 90% of postmortem samples obtained within a five-year period of diagnosis (70). As for miR-7-5p, which has been described as regulator of α-synuclein, HiTmIR identified key components of the PI3K/AKT signaling pathway like AKT3 and GSK3B as direct target genes. Balanced regulation of this signaling pathway is crucial for neuronal cell proliferation, migration, and plasticity (71). In general, the proposed pipeline allows the identification of a large number of target genes for a single miRNA in several cellular pathways and offers the possibility to discover previously hidden parts of the complex regulation network for conserved miRNAs.

Although some of the work steps of HiTmIR such as the consensus prediction and the validation by reporter assay are already described in the literature, the entire protocol, i.e. the combination of computational and experimental techniques to a systematic pipeline, is novel. With this pipeline, a new web service was developed to facilitate (i) the rapid design of potential reporter plasmid inserts by automating the steps of finding and excluding already validated targets, (ii) the search for all annotated transcripts and 3′UTRs per gene and (iii) the search for canonical binding sites in selected targets in real-time. Moreover, we incorporated functionality to split 3′ UTRs at different user-defined sequence locations and to highlight cut sites of restriction enzymes as well as a list of restriction enzymes without a cut motif in the target. These features were extensively fine-tuned and tested to improve the practical usability for massively parallel reporter assays and to reduce time intensive manual labor as much as possible. To the best of our knowledge there is no comparable free available tool published to date.

We implemented a data warehouse storing validated target pathways as well as positive and negative target gene sets. Especially negative target genes are lacking in the literature. Of 9679 reported target gene associations for *H. sapiens* in the miRTarBase, 9357 (97%) are positive and only 322 (3%) negative. In turn this highlights that negative targets are to a large extent not reported. However, such negative results are essential for developing new target predictors. Another challenge is that reporter assay results in databases such as the miRTarBase often come from heterogenous sources. Each manuscript contained in miRTarBase validates on average 1.6 target genes. This might pose challenges in the training process of individual target prediction programs. Our highly standardized positive and negative data set thus represents a valuable source to train or evaluate miRNA target prediction programs.

To further improve the sensitivity of our approach, it could be useful to include the analysis of synergistic effects due to multiple binding sites in the target 3′UTRs. As further down-stream validation strategy, miRNA target pathways additionally could be examined in a tissue-specific context (72,73). Other future developments include the extension from two miRNAs to a multitude of miRNAs that co-regulate the same signaling cascade in a systemic manner and to consider the dynamics of regulatory processes by exploring quantitative regulatory signals over time. Moreover, the setup of HiTmIR can be broadened to a more holistic approach, e.g. through testing of non-canonical binding sites.

## DATA AVAILABILITY

All data shown is freely available. The LUHMES miRNA microarray data has been deposited at GEO using accession ID GSE135151.

## Supplementary Material

gkaa1161_Supplemental_Files
