# Supplementary material for: Validation of human microRNA target pathways enables evaluation of target prediction tools
Source: Nucleic Acids Res. 2020 Dec 10;49(1):127–44. doi: 10.1093/nar/gkaa1161 (PMC7797041; doi:10.1093/nar/gkaa1161)
Supplement: gkaa1161_Supplemental_Files [file gkaa1161_supplemental_files.zip › Supplemental_document_gkaa1161.docx]

**Validation of human microRNA target pathways ena-bles evaluation of target prediction tools**

**Online methods**

**Automated dual luciferase reporter assay:** For the automated dual luciferase reporter Assay 2-2,5x10^4^ HEK 293 T cells were seeded out per well of a 96-well plate (Eppendorf, Hamburg, Germany) by the liquid handling system epMotion 5075 (Eppendorf, Hamburg, Germany). Sterile PBS was pipetted in the cavities of the 96-well plate to minimize edge effects. The next day, the HEK 293 T cells were transfected with 50 ng/well reporter vector without or with 3’UTR and 200 ng/well pSG5 empty vector or pSG5-miR-34a expression plasmid in the appropriate combinations using PolyFect transfection reagent (Qiagen, Hilden, Germany) corresponding to manufacturer’s protocol by the liquid handling system. 48 hours after transfection, cells were lysed using the liquid handling system, the cell lysates were prepared according to the manual of the Dual-Luciferase® Reporter Assay System (Promega, Madison, USA) and measured with the GlowMax navigator microplate luminometer (Promega, Madison, USA). For analysis the luciferase activity of each wild type 3’UTR reporter construct co-transfected with miR-34a was normalized to the luciferase activity of the empty reporter vector co-transfected with miR-34a. The workflow of the automated dual luciferase reporter assay is depicted in Figure 3C.

**miRNA expression plasmid and reporter constructs:** The pSG5-miR-34a expression vector was generated by Eurofins Genomics containing the nucleotides 9151617-9151816 of chromosome 1 (Eurofins Genomics, Ebersberg, Germany). The pSG5-miR-7 expression vector (Eurofins Genomics, Ebersberg, Germany) contains the nucleotides 88611724-88612046 of chromosome 15. For miR-34a-5p target gene validation, the sequences of the 191 3’UTRs of the TNF-, TGFB-signaling and the PD related target genes were synthetized and the ^~^490 nt long inserts were cloned into the pMIR-RNL-TK vector using the SpeI, SacI restriction sites by Eurofins Genomics (Eurofins Genomics, Ebersberg). The modifications of this reporter vector with an additional Renilla luciferase and TK promotor are described in Beitzinger et al. (74). The respective 3’UTR sequences of the 191 tested reporter vectors are given in the Supplementary Tables 6 and 10. The 3’UTR sequences of *CREB1_1 mut, CREB1_2 mut, TNFSF14 mut, DNM1L_1 mut, DNM1L_2 mut, AKT2 mut, SMAD7 mut, BMP8B mut, SMAD2_1 mut, SMAD2_2 mut, TGFB2 and EP300 mut,* were synthetized, the miR-34a binding sites were replaced by NruI restriction sites and the ^~^490 nt long inserts were cloned into the pMIR-RNL-TK vector using the SpeI, SacI restriction sites. For miR-7-5p target validation, the sequences of the 160 3’UTRs of the PD related target genes were synthetized and the ~690 nt long inserts were cloned into the pMIR-RNL-TK vector (BGI, Shenzhen, China). For future applications of HiTmIR to the PD-related pathways a standardized 3’UTR bank of plasmids was generated and checked for validity using high-throughput sequencing. The bank allows to test further miRNAs with HiTmIR easily against the same set of genes for maximal comparability of results.

**Cell lines, tissue culture:** Lund human mesencephalic (LUHMES) cells were purchased from the American Type Culture Collection (ATCC) and transfected for GFP-expression. The cells were cultured as previously described by Scholz et al. (30) in flasks pre-coated with 50 µg/mL Poly-L-ornithin and 1 µg/mL Fibronectin. Cells were cultured in advanced DMEM/F12 (Life Technologies GmbH, Darmstadt, Germany) supplemented with 1% N2-Supplement, 2 mM L-glutamine and 40 ng/mL basic fibroblast growth factor. The human HEK 293T and SH-SY5Y cells were purchased from the German collection of microorganisms and cell cultures (DSMZ) and authenticated using STR DNA typing by the DSMZ. HEK 293T cells were cultured as described previously (31). SH-SY5Y cells were cultivated in DMEM (Life Technologies GmbH, Darmstadt, Germany) supplemented with 20% Fetal bovine serum (Biochrom GmbH, Berlin, Germany), Penicillin (100U/mL), Streptomycin (100 µg/mL). All cell lines were cultured for less than three months after receipt.

**Differentiation of LUHMES cells:** For differentiation of LUHMES cells towards dopaminergic neurons, cells were cultured in advanced DMEM/F12 (Life Technologies GmbH, Darmstadt, Germany) supplemented with 1% N2-Supplement, 2 mM L‑glutamine, 1 mM dibutyryl cAMP, 2 ng/mL GDNF and 1 μg/mL tetracycline. After 48 hours, cells were trypsinized and seeded with 7.5 x 10^4^ cell/cm² in pre-coated flasks. Every 48 hours after re-seeding, fresh differentiation medium was added to the cells for six days.

**Neurotoxin treatment and RNA isolation:** To induce a PD-like phenotype, LUHMES cells were treated with 10 µM 1-methyl-4-phenylpyridinium (MPP+; Sigma Aldrich, Munich, Germany) 6 days after initiation of differentiation for 48 hours. Control cells were supplemented with H_2_O.

For RNA-Isolation, cells were lysed by QIAzol Lysis Reagent (Qiagen, Hilden, Germany) and total RNA was isolated using the miRNeasy Mini Kit (Qiagen, Hilden, Germany) according to manufacturer’s protocol. To determine RNA integrity, Agilent RNA 6000 Nano assay was used with the Agilent 2100 Bioanalyzer instrument (Agilent Technologies, Santa Clara, CA, USA).

**Immunocytochemistry:** For immunocytochemistry staining of TH and D2R, LUHMES cells were cultured as described previously and seeded on pre-coated 8-well µ-slides (ibidi GmbH, Gräfelfing, Germany) with 7.5 x 10^4^ cells/cm^2^. Medium was exchanged 48 hours after re-seeding. The cells were fixed at day 6 of the differentiation using an ice-cold mixture of 70% methanol and 30 % acetone for 3 minutes at room temperature, washed twice in cold PBS and blocked in PBS containing 10 % fetal calf serum for 1 hour at room temperature. The primary antibodies were diluted in PBS containing 1 % bovine serum albumin and incubated at 4°C overnight. TH was stained using a polyclonal rabbit antibody (Cat# ab112, RRID: AB_297840, abcam, Cambridge, UK) and D2R was detected using a goat polyclonal antibody (Cat# ab32349, RRID: AB_2094849, abcam, Cambridge, UK). After washing twice more with PBS, the secondary antibodies goat anti-rabbit IgG (Cat# A21428, RRID: AB_141784, Invitrogen AG, Carlsbad, California, USA) and donkey anti-goat IgG (Cat# A11056, RRID: AB_142628, Invitrogen AG, Carlsbad, California, USA) were diluted in PBS with 1 % BSA, applied and incubated for 1 hour at room temperature in the dark. Then cells were washed twice in PBS, the nuclei were counterstained with 300 nM DAPI (Sigma Aldrich, Munich, Germany) for 4 min at room temperature, washed once more and mounted using ibidi mounting medium. Images were taken with a Leica TCS SP8 microscope (Leica Microsystems, Wetzlar, Germany) and analyzed using LAS X software (version 3.5.5.19976, Leica Microsystems, Wetzlar, Germany).

**miRNA Microarray:** miRNA expression profiles after MPP+ treatment in dopaminergic neurons were monitored by using Agilent miRNA Complete Labeling and Hyb Kit as well as Agilent SurePrint G3 Human miRNA 80x60K Microarrays (Cat. No. G4872A, miRBase release 21.0, Agilent Technologies, Santa Clara, CA, USA) following the manufacturer's instructions and as described previously (32). For miRNA microarray, 100 ng total RNA was applied for the labeling reaction. First, RNA was dephosphorylated by calf intestinal phosphatase for 30 minutes at 37 °C and subsequently denaturated by using 100 % dimethyl sulfoxide (DMSO) for 10 minutes at 100 °C. Afterwards, RNA was labeled with Cyanine3-pCp by T4 RNA Ligase for 2 h at 16 °C, followed by hybridization of RNA to the microarray for 20 h at 55 °C with 20 rpm rotation in the SureHyb chambers (Agilent Technologies, Santa Clara, CA, USA). The miRNA microarrays were washed two times and dried before scanning on the Agilent Microarray Scanner G2565BA (Agilent Technologies, Santa Clara, CA, USA). Analysis of the received fluorescence signals was done by using Agilent AGW Feature Extraction software (version 10.7.1.1, Agilent Technologies, Santa Clara, CA, USA). Normalization of background corrected values was performed using GeneSpring by biological significance analysis (version 14.9, Agilent Technologies, Santa Clara, CA, USA). For further analysis, we considered only microRNAs that were detected in all of the tested samples. Statistical significance of differential expressed microRNA in MPP+ treated LUHMES was analyzed by paired t-test. The P-Values were adjusted for multiple testing by the Benjamini-Hochberg method. Fold change was calculated by normalization of the expression values to the mean expression value of the control samples. The raw microarray data has been deposited at GEO database (GSE135151).

**Western blot:** For western blot analysis of JNK3, SMAD2, SMAD7, CREB1 TH, CLOCK, and GRIA4 4.5x10^6^ SH-SY5Y cells per well of a six well plate were seeded out. After 24 hours the cells were transfected for miR-34a-5p over-expression with the allstars negative control (ANC) or with hsa-miR-34a-5p miScript miRNA Mimic (MIMAT0000255: 5'UGGCAGUGUCUUAGCUGGUUGU) and for miR-34a-5p inhibition with miScript Inhibitor Negative Control or anti-hsa-miR-34a-5p miScript miRNA Inhibitor (MIMAT0000255: 5 'UGGCAGUGUCUUAGCUGGUUGU) using HiPerFect transfection reagent (Qiagen, Hilden, Germany) according to the manufacturer’s protocol. 48 hours after transfection the cells were lysed using 2x lysis buffer (130 mM Tris/HCl, 6% SDS, 10% 3-Mercapto-1,2-propandiol, 10% glycerol) and sonicated on ice three times for three seconds. 10 µg of whole cell lysate were separated in a 5-15% TGX gel (Bio-Rad Laboratories Inc., Hercules, California, USA) and electroblotted on a nitrocellulose membrane (Whatman, GE Healthcare, Freiburg, Germany). JNK3 was detected by a monoclonal rabbit antibody (Cat# 2305, RRID: AB_2281744, Cell Signaling Technology, Danvers, USA), SMAD2 was detected by a monoclonal rabbit antibody (Cat# 5339, RRID: AB_10626777, Cell Signaling Technology, Danvers, USA), SMAD7 was detected by a polyclonal rabbit antibody (Cat# 42-0400, RRID: AB_2533512, Thermo Fisher Scientific, Rockford, USA) and CREB1 was detected by a monoclonal rabbit antibody (Cat# 4820, RRID: AB_1903940, Cell Signaling Technology, Danvers, USA). CLOCK was detected by a monoclonal rabbit antibody (Cat# 5157, RRID: AB_10695411, Cell Signaling Technology, Danvers, USA), TH was detected by a polyclonal rabbit antibody (Cat# 2792, RRID: AB_2303165, Cell Signaling Technology, Danvers, USA), GRIA4 was detected by a monoclonal rabbit antibody (Cat# 8070, RRID: AB_10829469, Cell Signaling Technology, Danvers, USA) and PARK2 was detected by a polyclonal rabbit antibody (Cat# 2132, RRID: AB_10693040, Cell Signaling Technology, Danvers, USA). β-actin and GAPDH served as loading controls and were detected with a monoclonal antibody against human GAPDH (Cat# 2218, RRID: AB_561053, Cell Signaling Technology, Danvers, United States) and an anti-β-actin monoclonal mouse antibody (Cat# A1978, RRID: AB_476692, Sigma Aldrich, Munich, Germany), respectively. All secondary antibodies were purchased from Sigma Aldrich (Sigma Aldrich, Munich, Germany). Quantification of the western blots was carried out with Image Lab Software Version 5.2.1 (Bio-Rad Laboratories Inc., Hercules, California, USA).

**Quantitative real-time PCR (qRT-PCR):** MiRNA microarray analysis and overexpression of miR-34a-5p in SH-SY5Y was verified by qRT-PCR. For miR-34a-5p overexpression, cells were lysed 48h after transfection by QIAzol Lysis Reagent (Qiagen, Hilden, Germany) and total RNA was isolated using miRNeasy Mini Kit (Qiagen, Hilden, Germany) according to manufacturer’s instructions. 150 ng total RNA was applied for reverse transcription with the miScript RT II Kit (Qiagen, Hilden, Germany). qRT-PCR was performed using miScript Primer Assay for hsa-miR-34a-5p, hsa-miR-7-5p, hsa-miR-181a-3p, hsa-miR-134-5p, hsa-miR-129-5p, hsa-miR-129-1-3p, hsa-miR-335-3p, hsa-miR-106b-3p, hsa-miR-412-5p and Custom miScript Primer for hsa-miR-4284 (Qiagen, Hilden, Germany) and the StepOnePlus Real-Time PCR System (Applied Biosystems, Foster City, United States) following the manufacturer’s protocol. RNU6B (Qiagen, Hilden, Germany) served as endogenous control. Statistical significance of differential expressed miRNAs in MPP+ treated LUHMES as well as miR-34a-5p over-expression was analyzed by paired, two-tailed t-test.

**Automated reporter assay construct generation using miRTaH:** To facilitate the straightforward generation of many hundred reporter assays we implemented miRTaH (miRNA Target assay Helper). Our web-application provides binding site matching, restriction site analyses, and supports selection and modification of target sequences. Moreover, miRTaH automatically generates uniform downloadable summary reports, which contain the selected parameters and modified sequences. As input, at least one miRNA and at least one target gene are required. If multiple miRNAs and target genes are provided the computations are performed for all combinations. If multiple transcripts are available, users can select one or several of them for further analyses per miRNA and target gene. The next step is to select the binding site patterns (from 5-mers to >8-mers) and the number of mismatches (from 0 to 2). Binding sites are automatically highlighted in the target 3’UTR. From 908 restriction enzymes, miRTaH selects those that have matching sites in the 3’UTR and that respectively don’t have sites in the 3’UTR. Having selected the matching restriction enzymes, users can download the results as PDF, CSV, or XLS spreadsheet. Further, the results can be copied to the clipboard. Also, the link can be stored and exchanged with other researchers for later use or exchange of information. As organisms, our web service supports *H. sapiens* and *M. musculus*. miRTaH is freely available online (<https://www.ccb.uni-saarland.de/mirtah>).

**Gene set over-representation analysis:**

Enrichment analyses were performed with GeneTrail2 ([https://genetrail2.bioinf.uni-sb.de](https://genetrail2.bioinf.uni-sb.de/)) using as input a list of predicted target genes for a so-called over-representation analysis (ORA). All computations were performed using standard parameters of GeneTrail2, except for the minimal and maximal sizes of resulting gene sets, which were set as 5 and 150, respectively. Gene Ontology, KEGG, Reactome, WikiPathways, and Reactome were used as target databases and p-values corrected using the Benjamini-Hochberg procedure setting an adjusted p-value cut-off at alpha-level 0.05. All other computations have been performed with R version 3.6.3.

**Supplemental Information**

**Supplemental Table 1:** qRT-PCR results for 10 selected miRNAs in MPP+ treated LUHMES cells.

**Supplemental Table 2:** miRWalk 2.0 consensus target predictions for miR-34a-5p.

**Supplemental Table 3:** GeneTrail2 enrichment (ORA) results for predicted targets of miR-34a-5p.

**Supplemental Table 4:** Target genes in selected miR-34a-5p target pathways.

**Supplemental Table 5:** Prediction results for miR-34a-5p on the pathway-level for selected disease pathways and prediction tools from miRWalk 2.0.

**Supplemental Table 6:** Compilation of 3’UTR target plasmids for miR-34a-5p tested by reporter assays.

**Supplemental Table 7:** miRWalk 2.0 consensus target predictions for miR-7-5p.

**Supplemental Table 8:** GeneTrail2 enrichment (ORA) results for predicted targets of miR-7-5p.

**Supplemental Table 9:** Target genes in selected miR-7-5p target pathways.

**Supplemental Table 10:** Compilation of 3’UTR target plasmids for miR-34a-5p tested by reporter assays.

**Supplemental Table 11:** Results of control reporter assays for miR-34a-5p.

**Supplemental Table 12:** Results of control reporter assays for miR-7-5p.

**Supplemental Table 13:** HiTmIR target reporter assay results for miR-34a-5p.

**Supplemental Table 14:** Binding site knockout reporter assay results for miR-34a-5p.

**Supplemental Table 15:** HiTmIR target reporter assay results for miR-7-5p.

**Supplemental Table 16:** Validation rate matrix for all miRNAs and target pathways / gene sets.

**Supplemental Table 17:** qRT-PCR results for miR-34a-5p in SH-SY5Y cells.

**Supplemental Table 18:** Western blot results for selected targets of miR-34a-5p using miRNA mimics.

**Supplemental Table 19:** Western blot results for selected targets of miR-34a-5p using miRNA inhibitors.

**Supplemental Table 20:** Results of short versus long 3’UTR assay results for selected targets of miR-34a-5p.

**Supplemental Table 21:** Performance evaluation results for prediction tools contained in miRWalk 2.0 and mirDIP 4.1 using the validation data set generated with HiTmIR.

**Supplemental Table 22:** Rule sets of target prediction tools obtained from association mining analysis of predicted and validated targets.

**REFERENCES**

74. Beitzinger, M., Hofmann, L., Oswald, C., Beinoraviciute-Kellner, R., Sauer, M., Griesmann, H., Bretz, A.C., Burek, C., Rosenwald, A. and Stiewe, T. (2008) p73 poses a barrier to malignant transformation by limiting anchorage-independent growth. *The EMBO Journal*, **27**, 792-803.
